# Light and shadows of a new technique: is photon total-skin irradiation using helical IMRT feasible, less complex and as toxic as the electrons one?

**DOI:** 10.1186/s13014-018-1100-4

**Published:** 2018-08-29

**Authors:** Michela Buglione, Luigi Spiazzi, Mauro Urpis, Liliana Baushi, Rossella Avitabile, Nadia Pasinetti, Paolo Borghetti, Luca Triggiani, Sara Pedretti, Federica Saiani, Alfredo Fiume, Diana Greco, Stefano Ciccarelli, Alessia Polonini, Renzo Moretti, Stefano Maria Magrini

**Affiliations:** 1 Radiation Oncology Department, University and Spedali Civili Hospital – Brescia, P.le Spedali Civili 1 –, 25123 Brescia, Italy; 2 Medical Physics, Spedali Civili Hospital – Brescia, P.le Spedali Civili 1 –, 25123 Brescia, Italy

**Keywords:** Total skin irradiation, Total skin electrons beam irradiation - TSEBI, Photons, Radiotherapy, Bone marrow, Primary cutaneous lymphoma (PCL), Toxicity

## Abstract

**Background:**

Radiotherapy is one of the standard treatments for cutaneous lymphoma and Total Skin Electrons Beam Irradiation (TSEBI) is generally used to treat diffuse cutaneous lymphoma and some cases of localized disease. Helical IMRT (HI) allows to treat complex target with optimal dose distribution and organ at risk sparing, so helical tomotherapy has been proposed as alternative technique to TSEBI but only one preliminary report has been published.

**Methods:**

Three patients treated (from May 2013 to December 2014) with Helical IMRT, with a total dose between 24 and 30 Gy, were retrospectively evaluated. Data about dosimetric features, response and acute toxicity were registered and analyzed.

Planned target coverage was compared with daily in vivo measures and dose calculation based on volumetric images used for set up evaluation as well.

**Results:**

The patients had a mean measured surface fraction dose ranging from 1.54 Gy up to 2.0 Gy. A planned target dose ranging from 85 to 120% of prescription doses was obtained. All doses to *Organs At Risk* were within the required constraints. Particular attention was posed on “whole bone marrow” planned V_10Gy_, V_12Gy_ and V_20Gy_ values, ranging respectively between 23 and 43%, 20.1 and 38% and 9.8 and 24%. A comparison with the *theoretical* homologous values obtained with TSEBI has shown much lower values with TSEBI.

Even if treatment was given in sequence to the skin of the upper and lower hemi-body, all the patients had anaemia, requiring blood transfusions, leukopenia and thrombocytopenia.

**Conclusion:**

Based on our limited results TSEBI should still be considered the standard method to treat total skin because of its pattern of acute and late toxicities and the dose distribution. In this particular case the better target coverage obtained with HI can be paid in terms of worse toxicity. Helical IMRT can instead be considered optimal in treating large, convex, cutaneous areas where it is difficult to use multiple electrons fields in relation with the clinical results and the limited and reversible toxicities.

## Background

Primary cutaneous lymphomas (PCL) are a group of heterogeneous diseases with a typical skin involvement and generally without systemic signs or symptoms, besides that the final stages of Mycosis Fungoides (MF) and Sèzary Syndrome (SS). MF/SS can be classified, according to TNMB classification, into four clinical stages depending on extension of skin involvement (in percentage of body surface), lymph node metastases, visceral involvement or presence of Sèzary cells in blood [1]. Age, type and disease extension, visceral metastases, increased LDH at diagnosis are recognized as prognostic factors [1–3].

Radiotherapy (RT) for PCL has been used in different clinical settings. In patients with solitary nodules of MF, or localized skin B/T disease, RT is the treatment of choice. The treatment of advanced-stage MF-SS is more complex and the final objective is to maintain clinical remission or stabilization of disease, improving quality of life.

Many studies report the efficacy of RT in patients featuring a set of localized lesions (up to three or four lesions covering less than 5% of the body). Routinely, these lesions are treated with a 6–9 MeV single direct electron beam, using a bolus to increase the dose to the cutaneous surface up to 95%. The standard prescribed dose varies from 30.6–36.0 Gy at 1.8–2.0 Gy per fraction. Recurrence rate is about 30% while 92% of patients obtain complete response [4–7].

Low dose large electron fields to treat the entire skin (Total Skin Electron Beam Irradiation, TSEBI) are used for advanced MF allowing a generalized and superficial treatment. [8, 9]. With the already known different standard techniques [10–13], however, it is extremely difficult to obtain a uniform dose distribution, considering the irregular shape of the human body surface [14].

Modern RT techniques have a goal not only to improve cancer cure rate, but also to reduce the treatment related adverse effects. Modern RT techniques have a proven capability to create highly conformal dose distributions, allowing the physicians to escalate the dose within the target volume and to spare adjacent organs at risk (OAR). The clinical use of Tomotherapy® induced radiation oncologists to use this technique to treat cutaneous circumferential localized lesions [15] and to think to use it to perform total skin irradiation. A preliminary report about the use of HI for total skin photon beam cutaneous irradiation has been already published [16]. We report a retrospective analysis on clinical/dosimetric data of three patients with diffuse cutaneous lymphoma treated with HI.

## Methods

From May 2013 to December 2014 three patients were treated with total skin photon radiotherapy. The first (#1) had MF (stage IVA1; T4N0M0); the second (#2) had diffuse cutaneous and systemic localizations of cutaneous T cell lymphoma; the third (#3) had a diagnosis of Granulomatous MF (stage II) (Table 1). After receiving adequate information about the technique and the possible acute and late effect of the treatment, all the patients accepted it and signed the informed consent.

**Table 1:** Patient’s features, RT prescriptions and response

	Patient #1 (female)	Patient #2 (male)	Patient #3 (male)
Diagnosis	MF (stage IVA1; T4N0M0) with erithrodermic disease,	Diffuse cutaneous and systemic localizations of cutaneous T cell lymphoma,	Granulomatous MF (stage II) slack skin
Previous treatments	Chemotherapy and UVB-PUVA, but never treated with radiotherapy	Different types of cutaneous therapies (PUVA), chemotherapy and localized RT, and finally proposed for palliative total-skin irradiation	Previously untreated
Prescribed dose (Median target dose)	27.0Gy/1.8Gy/fr (upper body) 26.0Gy/2.0Gy/fr (lower body) (22.05Gy/1.47Gy/fr Gy for the scalp and eyelids) 23 days split in between the two	28.8Gy/1.8Gy/fr (upper body) 28.8Gy/1.8Gy/fr (lower body) 15 days split in between the two	30.4 Gy/1.9 Gy/fr (upper body) 30.0 Gy/2 Gy/fr (lower body) 8 days split in between the two
Compensative electrons boost on “under-dosed” regions	One field electron boost (upper back) after the end of the photon treatment.	4 electron field boosts (right arm, left arm, inguinal, right foot dorsum). During the split between the first and the second part of the photon treatment	9 electron field boosts (right and left forearm, right and left arm, right back, left back, internal right thigh). During the split between the first and the second part of the photon treatment
Response to RT and duration	Short complete remission (6 months)	Short complete remission (6 months)	Complete remission (4 years)

### Immobilization and target definition

**Fig. 1:**
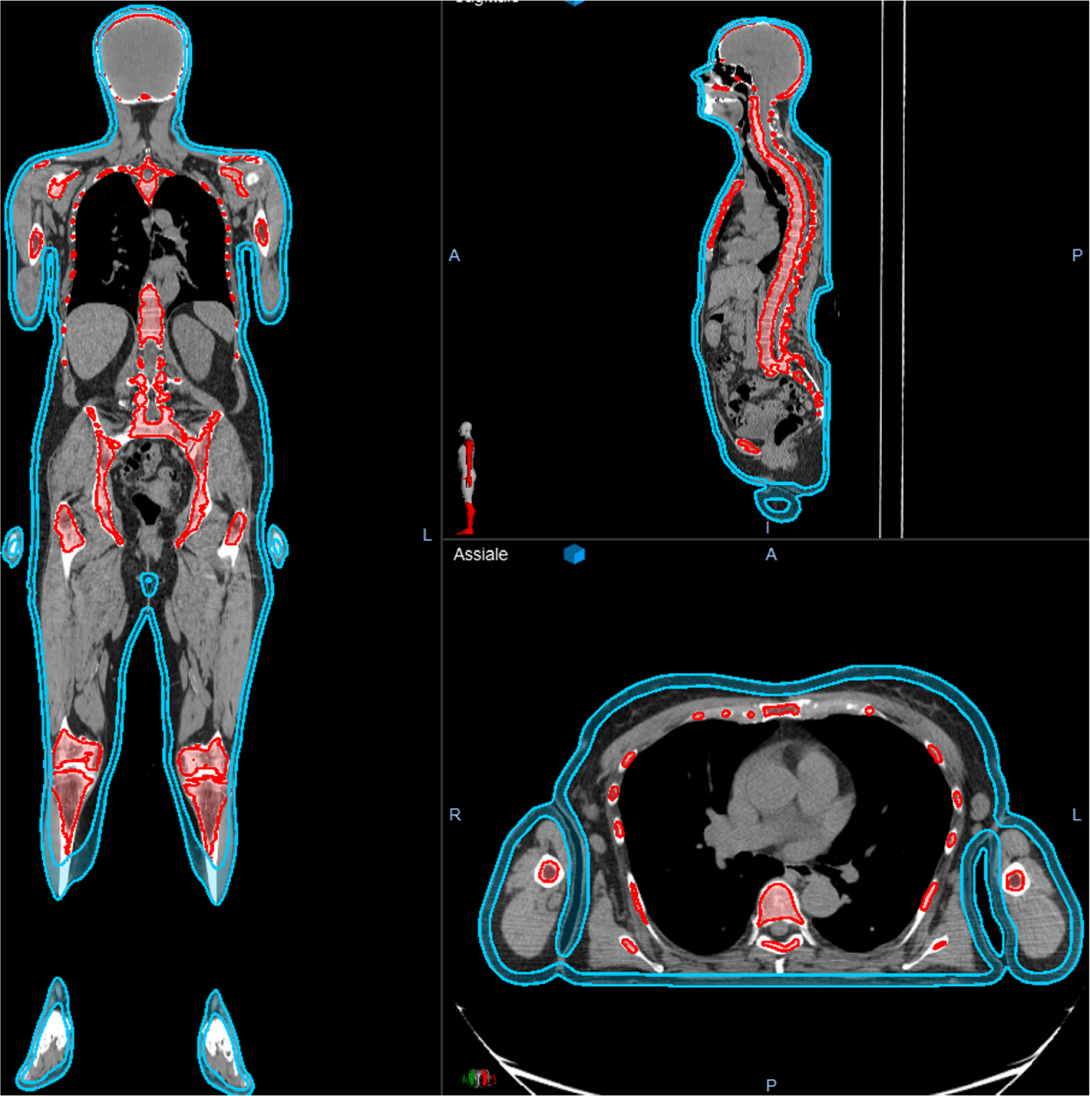
Target and bone marrow contouring

To obtain the better immobilization of the patient and treatment reproducibility, also in prevision of IGRT evaluations, set-up was obtained using vacuum-lock system and 5 points head & neck and abdominal thermoplastic masks. Gross tumor volume (GTV) included regions with evident disease (plaques). The *CTV-skin* included the entire body surface with a thickness of 5 mm. GTV plus 5 mm was included in CTV. PTV was defined as CTV plus 5 mm geometrical external margin and 7 mm internal margin. (Fig. 1). PTV was divided in PTV_eval (the intersection of PTV and the patient body contour) and OPTV, (PTV portion outside body contour).

Optic nerves, lens, eyes, lacrimal glands, cochlea, mandibles, parotids, thyroid, great vessels and heart, lungs, humeral heads, femoral heads, liver, stomach, intestinal cavity, spleen, kidneys, bladder, breast, rectum and uterus (for #1), testicles, penis and corpora cavernosa (for #2 and #3) were defined as OAR. Bone marrow was defined as the tissue within cortical bone of sternum, ribs, cranial, pelvic and long bones [17, 18].

The defined IGRT protocol included two daily MVCT, one at the beginning and one at the end of the volume in order to better evaluate the patient’s set-up along the all volume [25]. The median differences between the two MVCT revealed movements, were applied for the treatment.

**Fig. 2:**
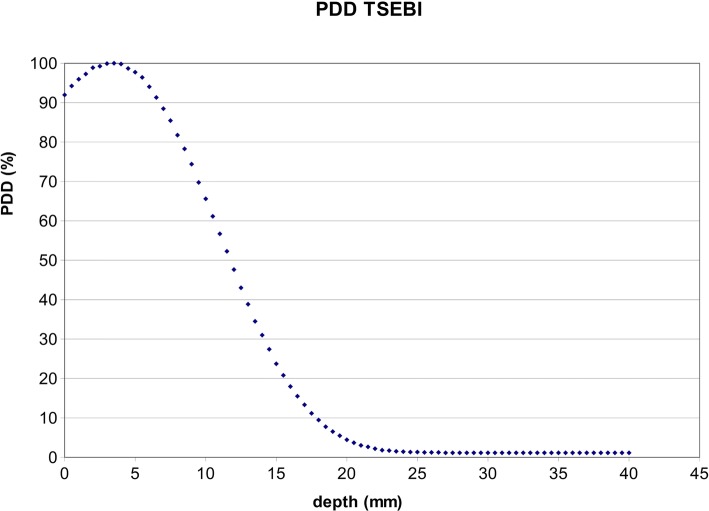
PDD of a TSBI electron beam

To compare bone marrow dose resulting from the use of different techniques (electrons versus photons), and even if with the limits of the absence of a TPS calculating the real electrons dose distribution, a theoretical planned DVH was retrospectively obtained considering the percentage depth dose (PDD) applied to patients treated with electrons (30.0 Gy; 86% at 7 mm). With the same intent, a bone marrow volume included in the superficial body layer was created. It was obtained with an automatic symmetrical contraction of the contoured external body volume of different extent (2 to 28 mm). Considering a dose prescription for TSEBI (28.8 Gy in 16 fraction), estimated DVH points were calculated, combining bone marrow volume included in the superficial layers and the measured dose curve in the conditions of the TSEBI setup. (Fig. 2).

### Clinical dose prescription

The prescribed doses were different for the three patients (Table 1) but always within the therapeutic range. Considering the 160 cm maximal target length in Tomotherapy® and to allow partial bone marrow recovery, the treatment was always delivered in two consecutive sessions of 13–16 fractions each. Upper body and lower body were treated posing the junction in a stable section of the body depending on each patient anatomy and set-up. The treatments were delivered 5 days a week for all the patients and all the sessions. All the patients had electron boosts to compensate under dosages (Table 1) or to treat macroscopic disease. In order to avoid over/under dosage in the treatments junctions, both the upper and the lower regions had two steps gradient regions, 5 cm each.

### Physical features of the treatment

The treatments were delivered with Helical IMRT (Tomotherapy®) with MVCT for set-up verification. All the plans were calculated with a 5 cm collimator. To reach a more homogeneous dose distribution, both PTV_eval and OPTV were divided in multiple sub-volumes during the optimization process. The OPTVs were included separately in the optimization to have a planned photon fluency that could expand beyond the planning CT body and treat efficiently the patients’ skin.

To reach adequate lungs and lens DVH values, the respective PRV were blocked during the optimization process. Other inner ad hoc volumes, obtained as a contraction of 2 cm of the body contours, were created and completely blocked in the optimization process to avoid internal organ irradiation. Eye Lens with 6 mm margin were, as well, completely blocked. All plans were elaborated with Helical Irradiation and computed with calculation grid fine. [19]. The plans characteristics of the different patients are reported in Table 2.

**Table 2:** Tomotherapy® plan characteristics

*Pat*	*Treatment session*	*Plan*	*Duration (min)*	*N. rotations*	*N. fractions*	*Number of PTV sub-volumes used in optimization*	*Number of region at risk used in optimization*
#1	Upper Hemibody	#1 U1	22.6	45.3	3	56	33
		#1 U2	22.6	43.7	12	56	25
	Lower Hemibody	#1 L1	23.6	19	10	14	14
		#1 L2	23.6	19	3	14	14
#2	Upper Hemibody	#2 U1	27.1	56	8	59	27
		#2 U2	28.9	56	8	68	28
	Lower Hemibody	#2 L1	40.0	50.1	3	45	35
		#2 L2	40.0	50.1	13	46	34
#3	Upper Hemibody	#3 U1	27.2	52.7	3	61	45
		#3 U2	27.2	52.7	13	74	56
	Lower Hemibody	#3 L1	29.4	83.9	15	71	42

All plans underwent three different kinds of Quality Assurance (QA) procedures: a) multiple standard pre-treatment dosimetric QA with measurements of the dose delivered to two phantoms (A1SL Exradin -Standard Imaging ionization chamber- in a cheese phantom; PTW Octavius chambers array for planar doses); b) daily delivered dose calculations based on MVCTs, co-registered with the planning CT (DODA, Dose of the day); c) in vivo *dosimetry* performed with Gafchromic films (EBT3®).

The Gaf-chromic films were positioned on the patients’ skin, in points considered at higher risk for dose distribution alterations. Lower and upper posterior aspect of torso, lateral aspect of the right and left arm, forehead, cheek, nape, scapula, sternum, right and left thigh, right and left hand dorsum, right and left foot dorsum, abdomen, pubic region, skin over fibula and medial/lateral aspect of right and left tibia were considered.

The exact positions of Gaf dosimeters were recorded to estimate, from daily dose distribution, the expected doses to match the result with. The films were handled according to AAPM TG-55 report [20].

### Response assessment

Patients were evaluated weekly during the treatment, one time 1 month after the end of the treatment and then every 3 months. Complete response was defined as complete disappearance of cutaneous lesions. Disease recurrence was defined in a multidisciplinary team (radiation oncologist, dermatologist and hematologist).

### Toxicity evaluation

Acute toxicity was defined as cutaneous and haematological damage appearing during the treatment and within 3 months after the end of radiotherapy. Adverse events occurring more than 3 months after the end of radiotherapy were defined as late toxicity. All the toxic events were defined with the CTCAE.4 scale. The possible relationship between clinical toxicity and dosimetric parameters was evaluated. The follow up was continued for all the patients (every 3 months) after the end of the treatment to evaluate late toxicity and outcomes.

### Statistical analysis

Results were compared with the *z–test* when a sample of data was compared with a reference value. t*-Student test* was used for paired data when two samples of data were compared. The differences were considered statistically significant if *p* < 0,01 to account for the high number of tests performed.

## Results

### Target dose

Deviation of planning median dose from the prescribed one were within 3% and therefore considered acceptable.

The planned doses per fraction to the target were compared with the DODA calculated for each fraction. Dose-volume points did not result significantly different, but in the few cases where the average DODAs were significantly higher than the provisional ones (eg. for patient #2 average D2% of the “lower volume” 2.1 Gy (SD 0.05 Gy) vs 2.1 Gy; average D95% 1.1 Gy (SD 0.06 Gy) vs 0.9 Gy; average D2% of the “upper volume” 2.3 Gy (SD 0.03 Gy) vs 2.2 Gy). Differences between the in vivo measures and DODA mean values for the different regions of the three patients were statistically different only in selected sites (upper back, right hand and foot dorsum for patient #1; the back and forearms for patient #2; the back, shoulders, harms and forearms for patient #3). Table 3 reports the target DVH outcomes for the patients obtained by means of Tomotherapy treatment planning. Cumulative plans are considered for each patient’s session. The results are calculated for the PTV_eval contours. The data show that the best target coverage was obtained in patient #3.

**Table 3:** Target dose volume points

	Target DVH points (Gy)			
Treatment session	DVH point	Patient #1	Patient #2	Patient #3
Upper hemi body: #1: 27/1.8 Gy #2: 28.8/1.8 Gy #3: 28.8/1.8 Gy	D (10 ml)	30.8	34.1	35.7
	D (2%)	30.0	33.1	34.9
	*D (50%)*	*26.4*	*28.5*	*30.9*
	D (90%)	22.4	22.2	23.8
	D (95%)	19.4	19.7	19.8
Lower hemi body: #1: 26/2 Gy #2: 28.8/1.8 Gy #3: 27.0/1.8 Gy	D(10 ml)	29.6	33.6	35.3
	D (2%)	28.9	33.0	34.5
	*D (50%)*	*25.7*	*28.5*	*30.9*
	D (90%)	23.1	19.7^a^	28.5
	D (95%)	21.7	14.2^a^	27.5

^a^electron field boost performed in the under dosage regions

### Organs at risk dose

Doses to the different OARs were within the safe limits. Higher mean doses were evident in superficial organs as lacrimal glands in pat #3 (16.7 Gy) parotids in all patients (range 18.1–20.9 Gy), thyroid (range 7.5–17.5 Gy), testicles respectively 27.0 Gy and 18.7 Gy for pat #2 and #3. The doses of all defined OAR are reported in Table 4.

**Table 4:** Previsional OAR’s mean doses and *DODA average mean doses CL’s (99,7% 3 σ)*

*Organs*	*Plans total mean dose (Gy)*			*DODAs mean doses CL – 3σ(Gy)*		
	Pat #1	Pat #2	Pat #3	Pat #1	Pat #2	Pat #3
Bowel (abdominal cavity)	1.1	10.9	3.9	0.89–0.93	8.7–9.0	4.6–4.8
Brain	4.5	3.2	4.6	4.3–4.5	3.1–3.6	4.5–4.6
Cord	3.1	6.7	6.1	3.2–3.6	6.69–6.73	6.1–6.9
Oesophagus	3.6	8.1	5.9	3.6–3.7	7.9–8.0	5.7–5.8
Heart	3.3	4.6	5.4	3.26–3.30	4.55–4.59	5.4–5.5
Lacrimal glands	3.1	3.3	16.7	3.9–4.6	3.7–3.8	13.3–13.6
Lens	1.9	2.2	5.9	2.1–2.7	2.2–2.4	6.2–6.5
Liver	2.4	4.3	4.3	2.3–2.4	4.2–4.3	4.4–4.5
Lungs	2.3	3.5	3.1	2.17–2.20	3.36–3.38	3.30–3.32
Parotids	20.9	19.5	18.1	19.5–20.3	16.7–18.2	18.5–18.7
Thyroid	17.5	14.4	7.5	14.1–14.7	14.5–14.7	6.9–7.0
Oral cavity	5.5	2.7	2.5	5.2–5.5	2.69–2.72	2.36–2.42
Spleen	2.7	4.6	9.9	2.5–2.6	4.3–4.6	9.4–9.6
Kidneys	3.1	3.8	3.3	3.0–3.2	3.56–3.58	3.34–3.39
Stomach	2.4	2.1	2.1	1.6–1.6	1.96–1.98	2.07–2.11
Bladder	11.0	2.6	1.4	9.1–9.4	2.30–2.34	1.31–1.31
Rectum	15.9	4.8	1.6	13.6–13.8	4.6–4.8	1.44–1.46
Uterus	13.8	/	/	10.8–11.3	/	/
Testicles	/	27.0	18.7	/	27.2–28.8	19.9–20.2
Penis and corpora cavernosa	/	24.0	9.9	/	22.3–22.4	7.5–8.0

Some structures had a large mean dose variation between the three patients. Lacrimal glands, parotids and thyroid mean variation was due to patient anatomy, while bladder, rectum and bowel cavity mean doses where affected both by patient anatomy and by the choice of upper and lower plans overlapping regions.

The differences between the DODA to the single organs and the planned ones were not statistically significant. The mean doses to the internal OARs are all < 1.0 Gy per fraction; the confidence limit of organs DODAs mean doses are shown in Table 4. Organs generally received lower doses than the planned ones.

### Bone marrow dose

The median and mean whole bone marrow cumulative doses for the entire treatment (upper plus lower plan) were 4.0 Gy and 8.5 Gy for patient #1; 4.5 Gy and 10.1 Gy for patient #2 and 6.5 Gy and 12.0 Gy for patient #3. These average cumulative doses were given in two consecutive sessions. Therefore, while the fraction of the bone marrow included in the upper part of the body is receiving a dose, the lower part of the body (and the corresponding bone marrow fraction) is not; the reverse is also true.

**Fig. 3:**
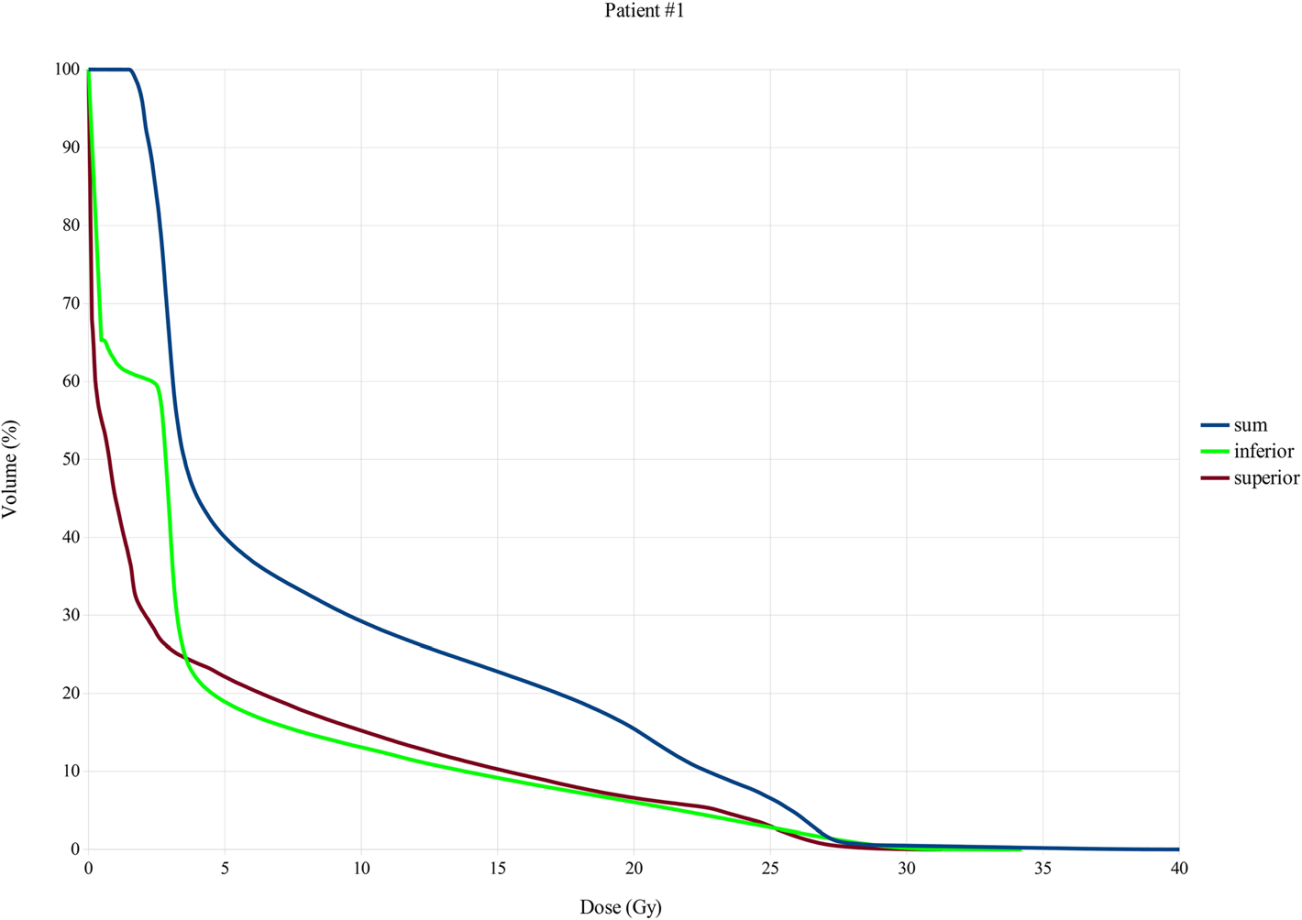
Bone marrow DVHs, Total, upper session and lower session DVHs patient 1

**Fig. 4:**
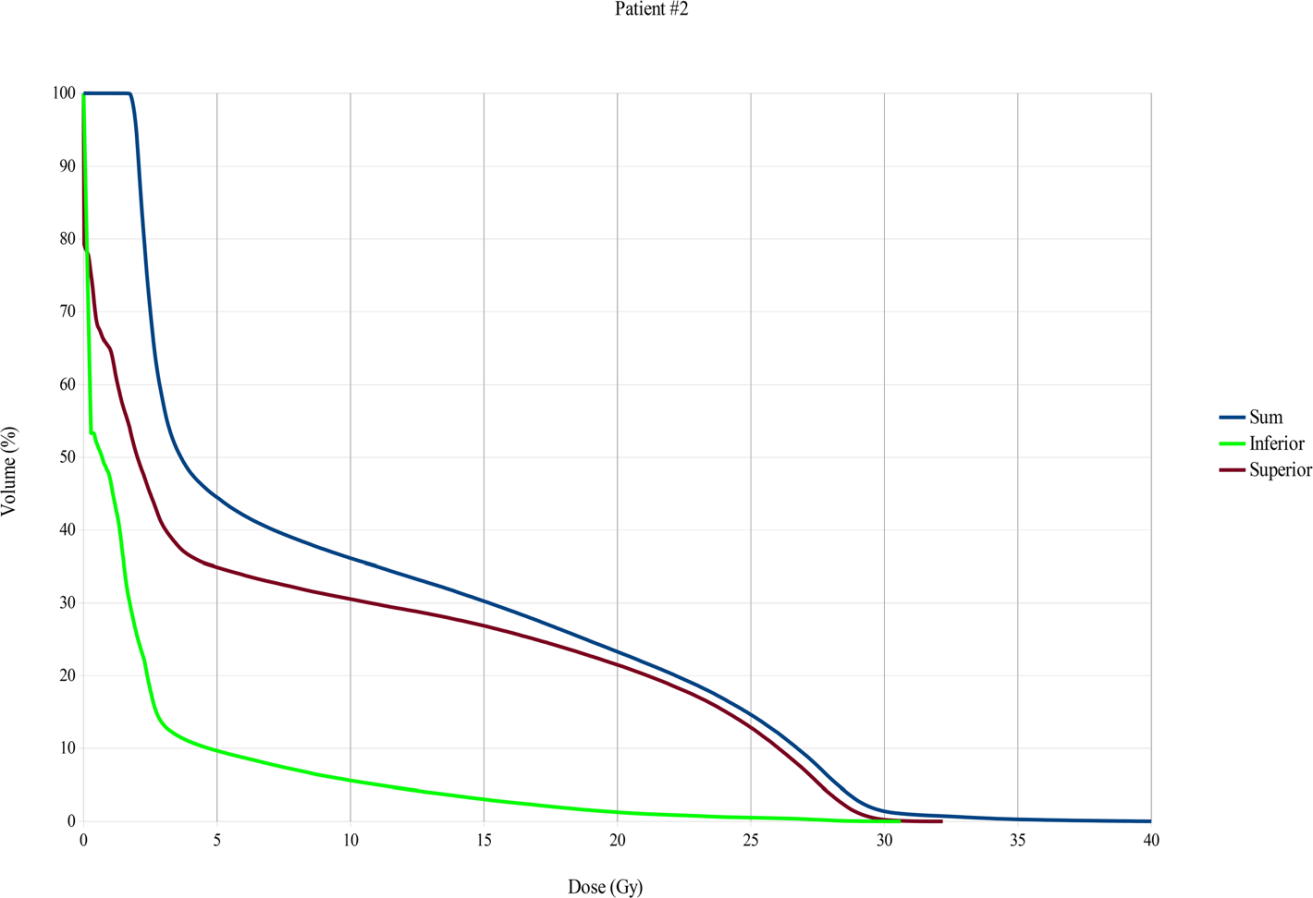
Bone marrow DVHs, Total, upper session and lower session DVHs patient 2

**Fig. 5:**
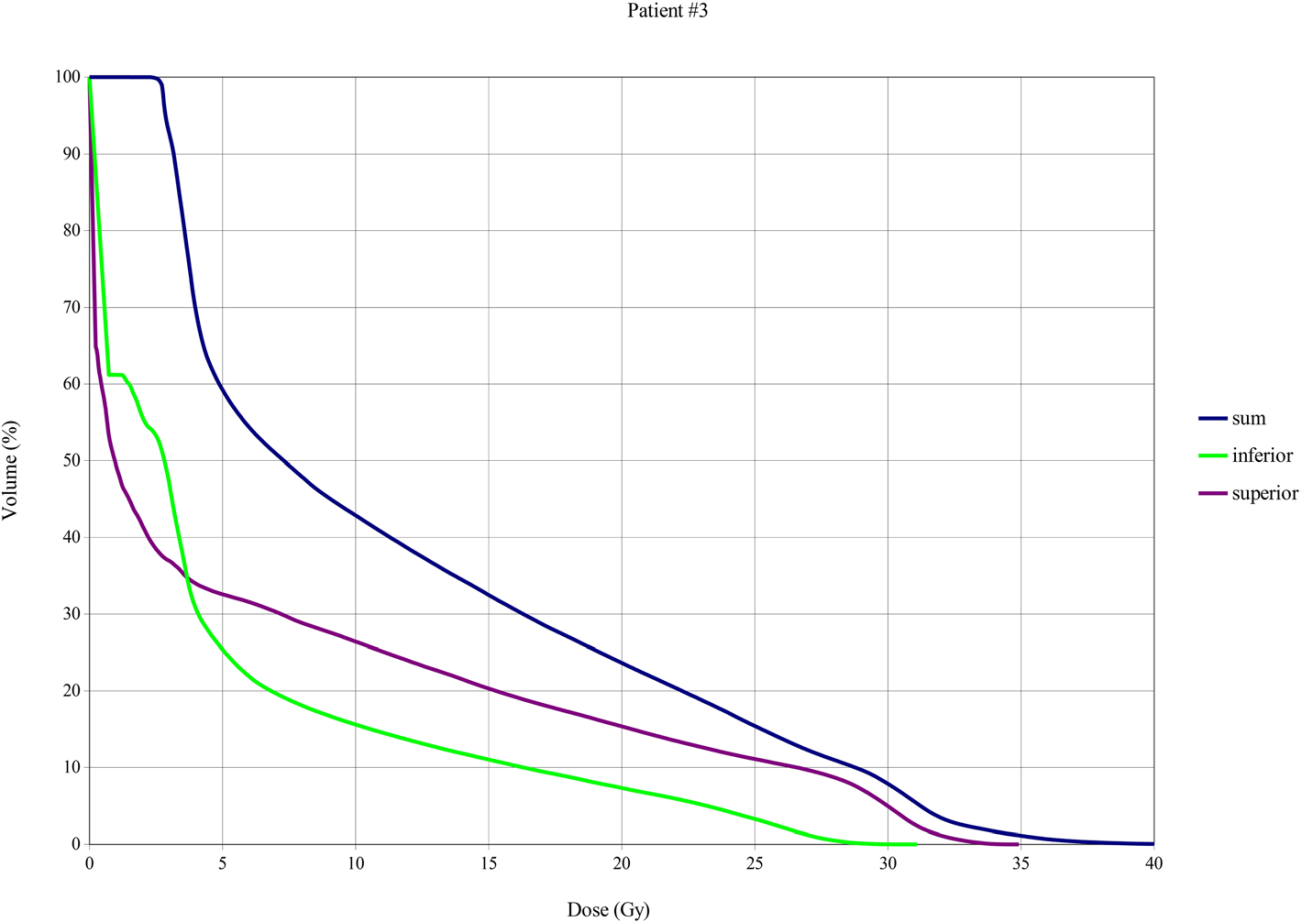
Bone marrow DVHs, Total, upper session and lower session DVHs patient 3

Whole bone marrow planned V_10Gy_, V_12Gy_ and V_20Gy_ values were 23%, 20% and 10% for patient #1; 37%, 34.1% and 23% for patient #2, 43%, 39% and 24% for patients #3. (Figs 3, 4, 5).

**Fig. 6:**
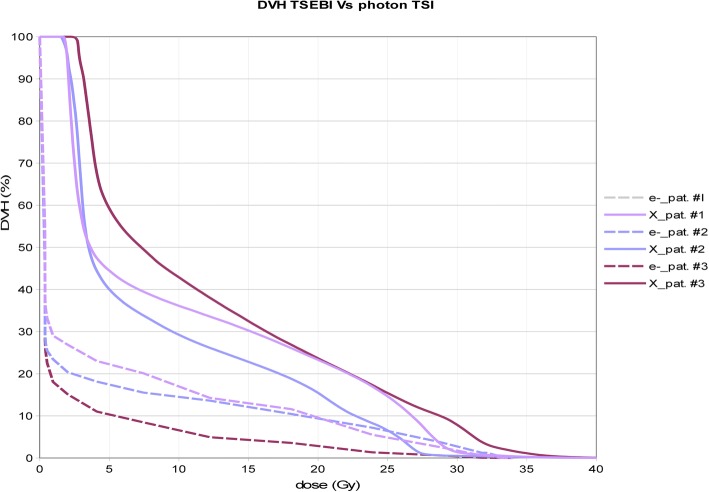
Bone marrow DVH’s. Solid lines photon beam TSI (planned cumulative doses); dotted lines (TSEBI cumulative doses as derived theoretically from PDD)

With TSEBI, the provisional values of bone marrow V_10Gy_, V_12Gy_ and V_20Gy_ were lower than those obtained with photons. In particular they were respectively 17%, 14.5%, 9.6% for patient #1; 14.5%, 13.7% and 9.4% for patient #2, 6.6%, 5.1% and 2.8% for patients #3. Median bone marrow dose for TSEBI would have been 0.4 Gy, 0.35 Gy and 0.35 Gy respectively for patient #1, #2 and #3; the average bone marrow dose would have been 3.6 Gy, 3.3 Gy and 1.3 Gy, respectively. (Fig. 6).

### Acute toxicity

#### Non-haematological toxicity

All patients had transient G2–3 skin toxicity with erythema and epitheliolysis, especially in sites with non-homogeneous dose distribution (axillary, inguinal regions and fingers). All patients experimented alopecia, nails alterations and oral mucositis (G2–3). All patients had plantar feet pain during 2 months after the end of treatment. All the symptoms were controlled with specific supportive care.

#### Haematological toxicity

All patients experienced anaemia requiring blood support and neutropenia requiring growth factors. Thrombocytopenia was evident in two patients and recovered within 6 months after RT. Haematological toxicity has been worse, including prolonged thrombocytopenia, in Patient #3. *Patient #1* had G2 anaemia twice, during the lower body and upper body treatment; G3 thrombocytopenia and G2 neutropenia. The haematological toxicity increased during the treatment in parallel with the increase of RT dose to the bone marrow. All the acute toxicities recovered within 2 months after the end of the treatment. *Patient #2* had G1 anaemia, G3 neutropenia and G1 thrombocytopenia at the end of both sessions, treated with blood transfusions and growth factors. Anemia, neutropenia and thrombocytopenia were respectively G2, G1 and G3 for *Patient #3* at the end of the treatment. All the acute toxicities but thrombocytopenia (70,000/ULt 3 years after the end of RT) recovered within 2 months after the end of the treatment.

### Response

The immediate disease response was good in all the patients. In one patient, treated with radical intent, the response was durable. (Table 1).

## Discussion

Large electrons fields perpendicular to the body of the patient, who is standing and rotated in 6 differently angled positions, are used to treat the entire skin, when indicated, according to the most used technique, the Stanford one [21]. The physical distribution of electrons [22] requires the use of fields perpendicular to the surface of the target and do not allow to treat concave or convex volumes with a homogeneous dose. With Total Skin Electron Beam Irradiation (TSEBI) a 2.0 Gy/fraction to the whole skin is delivered in 2 days (three body positions a day), and the patient is treated 5 days a week. No hematological toxicity is generally reported for TSEBI [23]. Nevertheless, this treatment is not considered *simple,* mostly for its duration and the need of adequate patient compliance. Additional complexity is due to difficulties in dosimetry and to the required equipment.

In recent years the use of sophisticated technologies allowed to treat targets close to OAR’s, and irregular volumes. There are some experiences suggesting the use of IMRT and Helical IMRT with IGRT to treat irregularly shaped body parts, like cutaneous circumferential targets on legs, arms, scalp or face [15, 24]. Image guided IMRT (also helical) is increasingly used to treat other complex targets, such as bone marrow in its entirety and the entire cranio-spinal volume [25–28].

The experience here reported revealed different critical issues using Tomotherapy® to treat a total skin volume. The problems are mostly related to treatment set-up, dosimetry, planning and verification, dose distribution to organs at risk, acute and late toxicity.

### Treatment set up

Patient’s compliance has to be adequate due to set up complexity (vac-lock, double thermoplastic mask, long daily treatment duration). Set-up and repositioning relevance has been emphasized also in other similarly demanding helical IMRT experiences, as total bone marrow-irradiation [29]. Not all the patients are able to reproduce and maintain the defined position, especially during the advanced phases of the treatment, when skin toxicity is more pronounced. This observation has been made also in the single, already published experience of total skin irradiation with helical IMRT, where the differences between the planning and the treatment position were about 3 mm, as measured during daily controls before and after each fraction [16]. Difficulties in the treatment set-up could be responsible of the statistically significant differences reported in the present paper between the planned, DODAs and gaf-measured doses during the treatment.

### Target coverage and homogeneity

The surface dose, reported in the unique already published experience of TSI with helical IMRT, is 84 cGy on the lesion (range 73.6 to 89.4 cGy) [16]. Our patients had a mean measured surface dose/fraction ranging between 1.54 Gy and 2 Gy. In general, we obtained a planned target dose ranging from 85 to 120% of prescription doses. Nonetheless, it was possible to identify hot and cold regions and to manage them; for this reason, re-planning was an important part of the process. This is clearly an advantage over the standard TSEBI technique approach, where it is not possible to correct for the inhomogeneous dose distribution [21].

### OAR dose distribution

The median doses to internal OARs, in the other published report, were 29.3 Gy and 24.7 Gy for the parotid and thyroid gland, respectively; 8.0 Gy for the brain; 5.2 and 6.8 Gy for liver and spleen; 3.8, 2.5 and 2.1 Gy for spinal cord, brain stem and lens, respectively. The authors report also median doses to lungs (4.5/4.7 Gy), heart (3.3 Gy) and kidneys (3.9/4.3 Gy) [16]. In our series, we reported mean organ doses and all the doses received by the OARs were largely within the accepted dose constraints and in line with those reported in the previous experience. As expected, doses to superficial organs were higher than those to the deeper ones, due to the photon characteristic depth curve.

### Bone marrow dose

Haematopoietic bone marrow is very radiation sensitive and it can be considered the most important OAR for this treatment because, particularly in districts as cranial bones, ribs and sternum, it is very close to the target (< 1 cm). Up to 30–40% of total bone marrow could be within the first 3 cm of the outer body layer. Bone marrow total dose was carefully analyzsed, also in relation with haematological toxicity and it was demonstrated a correlation with bone marrow RT dose.

In patients treated with radio-chemotherapy for cervical cancer, with a median pelvic RT dose of 45.0 Gy (range, 36.0–50.4) and a median *point A* dose (in patients undergoing intracavitary implants using low-dose-rate brachytherapy) of 85.0 Gy (range, 75.0–87.0), pelvic bone marrow V_10Gy_ values of > 90% are related to higher rates of hematologic G3-G4 toxicities and delays in chemotherapy administration [29]. Similarly, Albuquerque et al. reports, in patients who received 45.0 Gy with concurrent weekly cisplatin for cervical cancer, a significant increase of Grade 2–3 hematologic toxicity when ≥80% of whole pelvic bone received 20.0 Gy [30, 31]. The RT total body dose used to induce aplasia, conditioning bone marrow transplantation, is 12.0 Gy with a twice a day fractionation regimen (2.0 Gy/fraction) [31, 32].

Chen-Hsi Hsieh et al. identified the bone of the spine (cervical, thoracic, lumbar and sacrum) as *haematopoietic bone marrow.* Right and left iliac crest, right and left femurs, right and left pelvic bone, ribs and cranial theca, were not included in the bone marrow volume. The median doses reported in their experience ranged between 4 Gy (lumbar spine and sacrum) to 9 Gy (iliac crests), to > 12 Gy (femurs and pelvic bones). All these parameters can be used as a reference to evaluate bone marrow dosimetric results for the present series also considering that bone marrow was outlined in its entirety. The planned mean doses were 8.5 Gy for the first patient and, respectively, 10.1 and 12.0 Gy for patients #2 and #3. The average DODAs were slightly but significantly higher for the *first lower plan* and for the *second upper plan*, for the first patient and slightly lower for both the upper plans for the third patient. However, the skin of the upper and the lower hemi-body have been treated sequentially, with an interval up to about 3 weeks between the two treatment sessions. Thus, only about half of the bone marrow was directly exposed in each of the two sessions. It could therefore be considered that while part of the marrow is accumulating damage, the other is not (or not to the same extent) or is recovering.

The bone marrow mean and median doses are slightly higher for patient #3, probably in relation with treatment planning optimization. This could be the reason for protracted thrombocytopenia of patient #3, along with the shorter interval between the first and the second session of the treatment (Table 1). In fact, the total mean dose of 12.0 Gy was delivered during the entire treatment (31 fractions) with a mean dose per fraction of 0.4 Gy.

The average bone marrow doses given with photon TSI, even if are much lower than those used as a conditioning regimen in bone marrow transplantation, are sufficient to induce bone marrow toxicity. In fact, this is what happened with low dose TBI used in different trials to obtain bone marrow disease control in follicular lymphomas. [33, 34] The bone marrow dose volume points for a hypothetical TSEBI treatment, with the same dose regimen, would have been significantly lower for all patients. Since this is the main cause of hematological toxicity, it can be considered as a limiting factor for the use of this technique to treat total skin. In fact, one out of only three patients had late thrombocytopenia.

### Toxicity

As far as toxicity is concerned, doses to the internal organs (abdominal cavity, kidney, spleen and eyes) are lower than those defined as “constraints” but they are higher than those given using electrons that is less than 0.3 Gy on average to organs located at a depth of more than 3 cm. (Fig. 4).

Although a direct comparison between the toxicity related to HT and TSEBI is not possible, due to the very small number of HT treated cases, data from the literature (6) and from our experience with TSEBI point to a substantial similarity of the skin/nails damage and of the other non-hematologic toxicities induced by the two techniques. Superficial organs as lacrimal glands, thyroid, and lenses receive similar or lower doses than those given with TSEBI, and the resulting toxicity does not seem to be substantially different.

However, TSEBI hematologic toxicity is scarce, whereas all our patients had different degrees (G2–3) of hematologic toxicity (even if patient #1 marrow doses were close to those expected with TSEBI).

## Conclusions

Based on previous published experiences of the use of Helical IMRT to treat large and complex superficial volumes, this is the second report on total skin treatments with photons performed with this technique.

As expected, dose homogeneity has been better than with TSEBI and a durable remission has been obtained in the unique patients treated with radical intent.

Even if a relative increase in bone marrow toxicity was expected, its extent was unexpected, considering also the split course nature of the treatment.

Therefore, according to our results, TSEBI should still be considered the standard method to treat total skin because of its pattern of acute and late toxicities and further use of helical IMRT for this treatment discouraged: the better target coverage obtained with HI is not clearly related to better clinical response and can possibly induce worse toxicity.

On the other hand, considering the data published, the clinical results and the limited and reversible toxicities in treating volumes smaller than total skin, Helical IMRT can be considered optimal in treating large, convex, cutaneous areas where it is difficult to use multiple electrons fields.

## Abbreviations

CR: Complete response
CTV: Clinical target volume
DODA: Dose of the Day. Calculation of daily delivered dose based on the verification MVCT
DVH: Dose volume histogram
Dy%: Dose at least received from y% of volume (y is a number between 0 and 100)
GTV: Gross tumor volume
HI: Helical IMRT
IGRT: Image guided radiation therapy
IMRT: Intensity modulated Radiation Therapy
MF: Micosis Fungoides
MVCT: Megavoltage computed tomography
OAR: Organs at risk
OPTV: Outside the body surface PTV
PCL: Primary cutaneous lymphomas
PD: Progressive disease
PR: Partial response
PRV: Planning organ at Risk Volume
PTV: Planning target volume
QA: Quality Assurance
RT: Radiotherapy
SS: Sèzary Syndrome
TSEBI: Total skin electron beams irradiation
V_XGy_: Percentage of volume receiving at least X Gy (X is a number)
